# Simulation of prospective PIRCHE-II molecular matching in Canada: a feasibility study

**DOI:** 10.3389/fimmu.2026.1703762

**Published:** 2026-02-10

**Authors:** K. R. Sherwood, O. P. Günther, F. Fenninger, J. Tran, J. Lan, M. Niemann, R. Sapir-Pichhadze, P. A. Keown

**Affiliations:** 1 Department of Pathology and Laboratory Medicine, University of British Columbia, Vancouver, BC, Canada; 2 Günther Analytics, Vancouver, BC, Canada; 3 Department of Medicine, University of British Columbia, Vancouver, BC, Canada; 4 PIRCHE AG, Berlin, Germany; 5 Department of Nephrology and Medical Intensive Care, Charité Universitätsmedizin Berlin, Berlin, Germany; 6 Department of Medicine, McGill University and McGill University Health Research Institute (MU-HRI), Montreal, QC, Canada

**Keywords:** HLA compatibility, HLA epitopes, immunogenetics, kidney transplantation, molecular matching

## Abstract

**Introduction:**

Organ allocation to minimize Human Leukocyte Antigens (HLA) disparity between donor and recipient has been shown to improve outcomes but is limited by the enormous HLA diversity. PIRCHE-II in silico model considers the HLA peptide binding characteristics of recipients to quantitate molecular compatibility. Having previously published the feasibility of including B-cell eplets into simplified, simulated match algorithms, here we assess the feasibility of using PIRCHE-II epitope optimised allocation, in a cohort of ~1500 heterogenous renal patients and donors within the National Canadian organ transplant program.

**Methods:**

This test-bed-only analysis provides critical first steps to understanding if prospective matching is indeed feasible, in a Canadian transplant population.

**Results:**

Simplified base-case simulation models optimizing for PIRCHE-II score demonstrate that molecular matching across all 5 HLA gene loci (A, B, C, DR, DQ) is achievable in <10% of patients, and hence would not be realistic for clinical allocation. In contrast, molecular matching with a low PIRCHE-II score at the principal HLA class II DRB1 or DQB1 loci may be achieved in over 90% of patients compared with the base-case scenario.

**Discussion:**

In reality, the precise matching probability is governed by multiple factors including waiting-list size, donor organ factors, and other allocation restrictions (i.e. ABO blood type, presence of anti-HLA antibodies, clinical urgency), which would further impact match probability.

## Introduction

Transplantation is the optimal intervention for irreversible renal failure, demonstrating superior survival rates, enhanced quality of life, and cost-effectiveness compared to alternative treatments (1, 2). Despite impressive initial outcomes, with 1-year kidney graft survival rates frequently surpassing 95%, an important proportion of grafts fail within the first decade (3). While multiple factors compromise graft longevity, immune recognition of proteins encoded by the Human leukocyte antigen genes (HLA) leading to graft rejection has been the predominant cause of failure (4, 5). These HLA proteins, expressed on almost all nucleated cells, provide an exquisitely sensitive mechanism to discriminate self from non-self (foreign) components. HLA genes exhibit exceptional allelic diversity, creating a vast pool of possible HLA phenotypes (6). This polymorphism facilitates broad capabilities for antigen presentation, crucial for immune defense, but presents significant challenge in the context of transplantation.

Reducing the HLA disparity between a potential donor and recipient to optimize molecular compatibility improves outcomes in living and deceased donor transplantation (7–9) but is difficult to achieve due to the heterogeneity of the HLA gene region. Various approaches have been considered to increase molecular compatibility (10–18), and advances in HLA sequencing technologies and in silico models for predicting B- and T-cell epitopes have enabled the development of algorithms to characterize the degree of donor-recipient incompatibility at a more granular, molecular level. One computational approach used to assess HLA compatibility at the structural level is the PIRCHE *(Predicted Indirectly ReCognisable HLA Epitopes*) algorithm which considers putative donor HLA-derived peptides and predicts T-helper cell activation, critical for B-cell proliferation and affinity maturation in linked recognition (18, 19). By modelling the HLA-specific peptide binding motif using neural networks, PIRCHE predicts allo-peptides with a high probability of extracellular presentation by recipient HLA class II proteins. Such peptide-HLA complexes are potential targets for recipient T cells *(reviewed in* (20)*).* Each disparity is counted in developing a PIRCHE-II score, a higher score indicating an increasing number of unique distinct donor-derived allo-peptides presented by recipient HLA class II (19).

Retrospective studies have demonstrated the correlation of higher PIRCHE-II scores with adverse clinical outcomes and an increased risk of developing *de novo* donor-specific anti-HLA antibodies (dnDSA) (21, 22), graft rejection (22–24) and graft loss (21, 25, 26). PIRCHE-II is therefore employed clinically to inform post-transplant immune suppression and care. Only a small minority of patients and donors are closely matched under current allocation strategies, however, limiting potential clinical and societal benefit. We therefore enquired whether leveraging PIRCHE-II scores to inform organ allocation to optimize molecular compatibility could maximize benefit in most recipients. This novel application depends critically on the proportion of patients who can be closely matched in a given population. This crucial aspect remains largely unexplored, with just a single paper considering prospective PIRCHE-based deceased donor kidney allocation (27). We have previously reported a simulation model to determine the feasibility of donor organ allocation using HLAMatchMaker (v02/v02.2) eplets for patients awaiting renal transplantation (28). Here we explore (a) the feasibility of prospective organ allocation using single- or multi-gene PIRCHE-II T-cell epitope scores, (b) the influence of varying waitlist sizes and donor frequencies across Canadian provinces and (c) individual patient propensity for algorithmic matching.

## Materials and methods

### Patients and HLA gene sequencing

This nested Canadian population study included 1,411 subjects (1,150 patients assessed for renal transplantation and 261 deceased donors) who were genotyped by next-generation sequencing (NGS) between October 2016 and January 2019 at the Provincial Reference Immunology Laboratory, Vancouver, British Columbia (BC). Sample preparation and NGS for all 11 classical HLA genes were performed as described in our previous studies (29). Clinical characteristics and HLA allele carrier rates have previously been described (29). Research was approved by the University of British Columbia Clinical Research Ethics Board (#H22-01975).

### Descriptive statistics

The dataset was summarized providing the *n* value overall and for each group, with mean, median, range, and standard deviations for continuous variable and counts and proportions for categorical variables.

### Donor allocation rules

The baseline scenario was structured to approximate the current Canadian transplant model in which deceased donor allocation is performed primarily within the province of kidney origin and time spent on the waitlist is the principal determinant of rank order for transplantation. Allocation is generally constrained by ABO identity rather than compatibility to avoid detriment to blood group O recipients by allocating group O donor organs to other blood group recipients. Organs are normally allocated independent of HLA compatibility, which is employed to select between individuals of otherwise equal ranking. Priority rules which apply in a small proportion (~10%) of subjects who are highly sensitized or in urgent clinical need (e.g. children, loss of dialysis access, or other cases of exceptional clinical urgency) were not included in this first simulation exercise.

### PIRCHE-II analysis

The PIRCHE-II algorithm (version 3.3, IMGT database 3.40) (19) was used to count the number of donor-derived non-self HLA epitopes that are predicted to be presented in the peptide binding groove of recipient HLA-DRB1 molecules, denoted as the PIRCHE-II score (30). These scores were calculated individually for each of five HLA genes HLA-A, -B, -C, -DRB1, -DQB1, and combined for all 5 gene loci (PIRCHE sum) for each donor-recipient pair. PIRCHE-II molecular mismatch scores were recorded in a Match-List table for post-simulation analysis and were stratified into 4 clinically relevant categories of <9, ≥9 to <35, ≥35 to <90 and ≥90 molecular mismatches as defined by Lachmann et al. (21).

### Simulation strategy

A simulation framework for kidney allocation was implemented in R (MRAN 3.5.3) to model matching between deceased donors and kidney transplant candidates. A greedy algorithm was incorporated that paired each donor with the recipient having the lowest PIRCHE-II score at the relevant HLA locus or combination of loci, with the waiting time determining priority in cases of identical scores. Simulation was initialized using waitlists of specified size as described below, and bootstrapped transplant candidates were added one at a time until all kidneys were allocated. This process produced an initial rank ordering where the first candidate added was at the top, and the last candidate added was at the bottom of the waitlist. Each donor was considered to provide two kidneys for matching with candidates on the waitlist using defined allocation rules. Before the next donor was entered into the model, two new recipients were randomly selected from the candidate distribution and added to the bottom of the waitlist, maintaining a constant waitlist size over the course of the simulation, which has been identified as a crucial paradigm for stable histocompatibility in allocation simulation (27). All organ offers were assumed to be accepted at the time of availability and simulation continued until all donor kidneys were allocated within a virtual one-year period.

### Simulation cohorts

Allelic frequencies of recipients and donors were extrapolated to create a set of simulation cohorts consistent with Canadian transplant frequencies from the Canadian Institute of Health Information data (54). Exploratory waitlist cohorts ranging from 100 – 2,500 patients were developed to encompass individual provincial frequencies (ranging from 100–800 patients) and the total Canadian waitlist of over 2,000 patients. Deceased donor frequencies were related to these values to create waitlist patient-to-donor ratios ranging from 2:1 to 4:1, including the Canadian national ratio of approximately 2.6:1. A total of 175 sets of simulation were performed each with 10 replicates each (running the same simulation for different random orderings of recipients and donors) from which the cumulative probabilities of increasing mismatch scores were derived.

## Results

### Overall PIRCHE-II scores in base-case and prospective molecular matching scenarios

#### Base-case distribution of PIRCHE-II scores and dispersion

**Figure 1:**
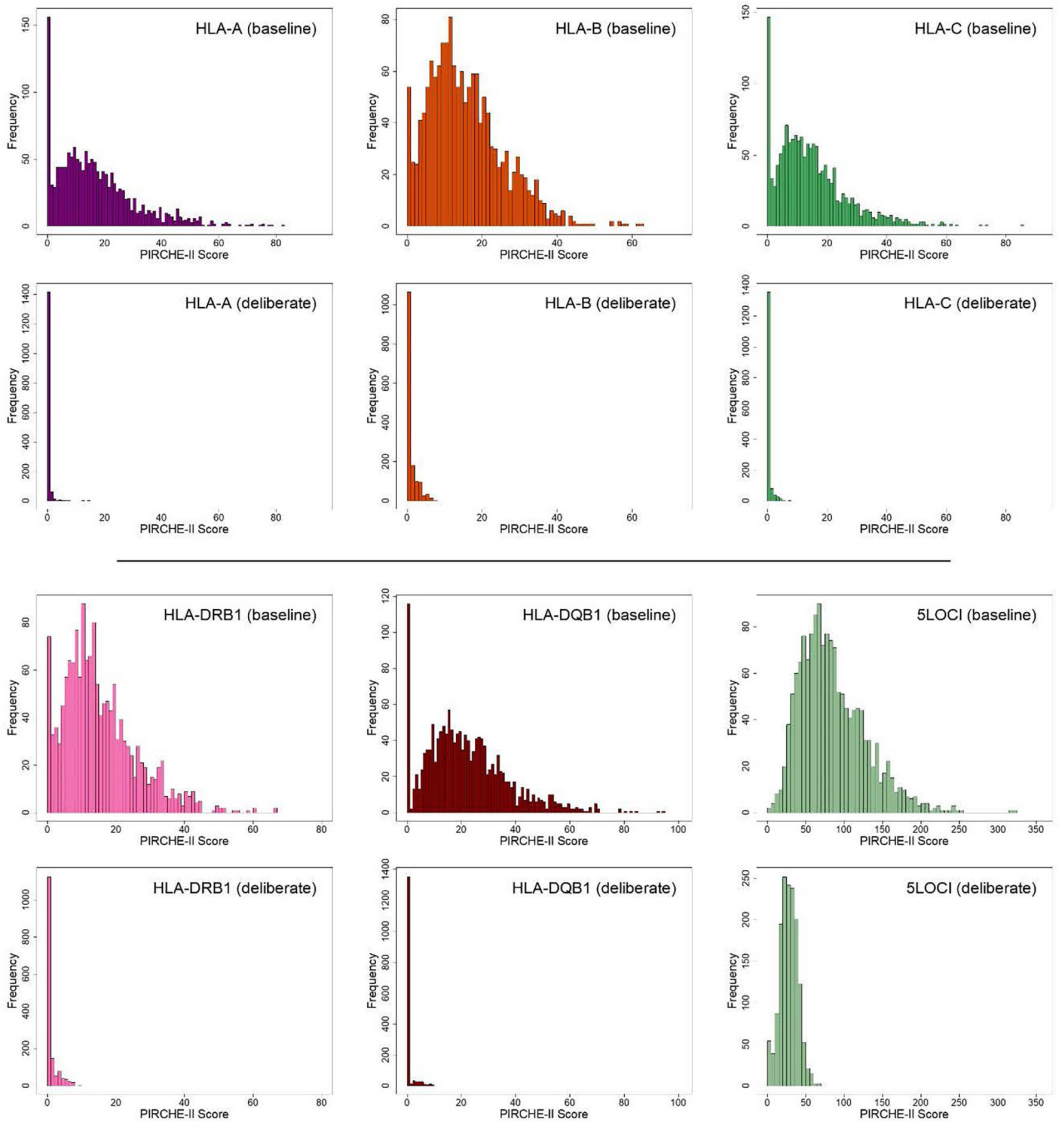
PIRCHE-II score distributions for individual HLA-A, B, C, (top panel) DRB1 and DQB1 loci and all 5LOCI combined (bottom panel) for 1,524 bootstrapped ABO identical recipient-donor match pairs in the base-case or deliberately matched scenarios, for a Canadian national waitlist.

Distributions of the 5 individual HLA locus-specific PIRCHE-II scores and the PIRCHE-II sum score (which includes all 5 HLA loci), are shown in **Figure 1**. Median PIRCHE-II scores (and ranges) were 15 (0-83) for HLA-A, 14 (0-63) for HLA-B, 13 (0-86) for HLA-C, 14 (0-67) for HLA-DRB1, 20 (0-95) for HLA-DQB1, and 78 (0-323) for all 5 HLA genes (5LOCI). The probability of achieving a zero PIRCHE-II score was <8% for each individual locus, and 0.13% for the combination of all 5 HLA loci. The proportions (and absolute numbers) of zero-PIRCHE-II-score pairs were 0.0748 (n=114) for HLA-A, 0.0223 (n=34) for HLA-B, 0.0636 (n=97) for HLA-C, 0.0361 (n=55) for HLA-DRB1, 0.065 (n=99) for HLA-DQB1 and 0.0013 (n=2) for all 5 loci.

#### Molecular matching minimizes PIRCHE-II scores and dispersion

Simulation employed to explore the impact of organ allocation based on prospective molecular matching demonstrated an important reduction in median PIRCH-II scores and population dispersion at all gene loci as shown in **Figure 1**. Median PIRCHE-II scores (and ranges) were reduced to 0 (0-15) for HLA-A, 0 (0-8) for HLA-B, 0 (0-8) for HLA-C, 0 (0-10) for HLA-DRB1, 0 (0-13) for HLA-DQB1, and 28 (0-69) for all 5 gene loci. The probability of achieving a zero PIRCHE-II score increased considerably from <8% to <87% for each individual locus, and from 0.13% to 2.23% for the combination of all 5 HLA loci. The specific probabilities (and absolute numbers) were 0.8675 (n=1322) for HLA-A, 0.5892 (n=898) for HLA-B, 0.811 (n=1236) for HLA-C, 0.626 (n954) for HLA-DRB1, 0.8445 (n=1287) for HLA-DQB1 and 0.0223 (n=2) for all 5 loci.

#### Deliberate molecular matching for different waitlist sizes

**Figure 2:**
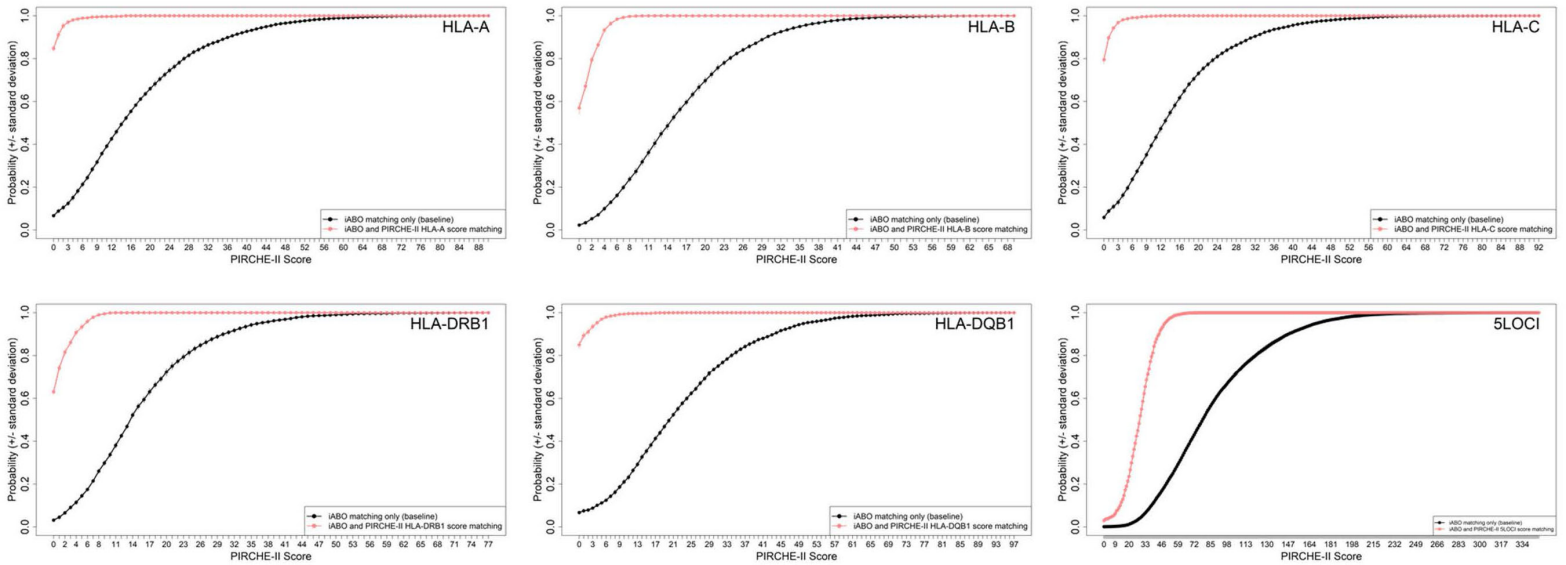
Simulated kidney allocation in ABO identical donor/recipient pairs. Figures show the probability of achieving a PIRCHE-II score when matching at individual HLA-A, HLA-B, HLA-C, HLA-DRB1 and HLA-DQB1 loci and all 5LOCI HLA genes combined for deliberate PIRCHE-II matching (red curves) and base-case blood group identical matching only (black curves) in kidney patients and deceased donors. Plots show Error bars are calculated as the standard deviation of 10 repeated simulation runs.

Scenario analyses performed across the range of provincial programs confirmed the importance of waitlist size in achieving higher proportion of patients with lower scores. Compared with no deliberate PIRCHE-II matching (**Figure 2**, black lines), the median score (and range) showed a reduction of PIRCHE-II score (**Figure 2**, red lines) from 14.7 (0-90) to 0 (0-15) for HLA-A, 15.0 (0-69) to 0 (0-12) for HLA-B 13.3 (0-92) to 0 (0-16) for HLA-C, 14.0 (0-77) to 0 (0-11) for HLA-DRB1, 20.5 (0-97) to 0 (0-23) for HLA-DQB1 and 79.25 (0-347) to 29.1 (0-77) for the 5LOCI combined (**Figure 2**).

### Ordinal PIRCHE-II scores in base-case and prospective molecular matching scenarios

#### Base-case distribution of categorical PIRCHE-II scores

The data above shows that a zero PIRCHE-II match is too demanding a target for a prospective strategy to optimize donor/recipient compatibility. In order to relax these criteria, we divided patients into 4 strata proposed by Lachman et al. (21). The base case scenario showed a probability of PIRCHE-II scores within each of the 4 strata of low (<9), elevated (≥9 to <35), intermediate (≥35 to <90) and high (≥90)).

#### Improvement in PIRCHE-II risk categories is highly influenced by waitlist size

**Figure 3:**
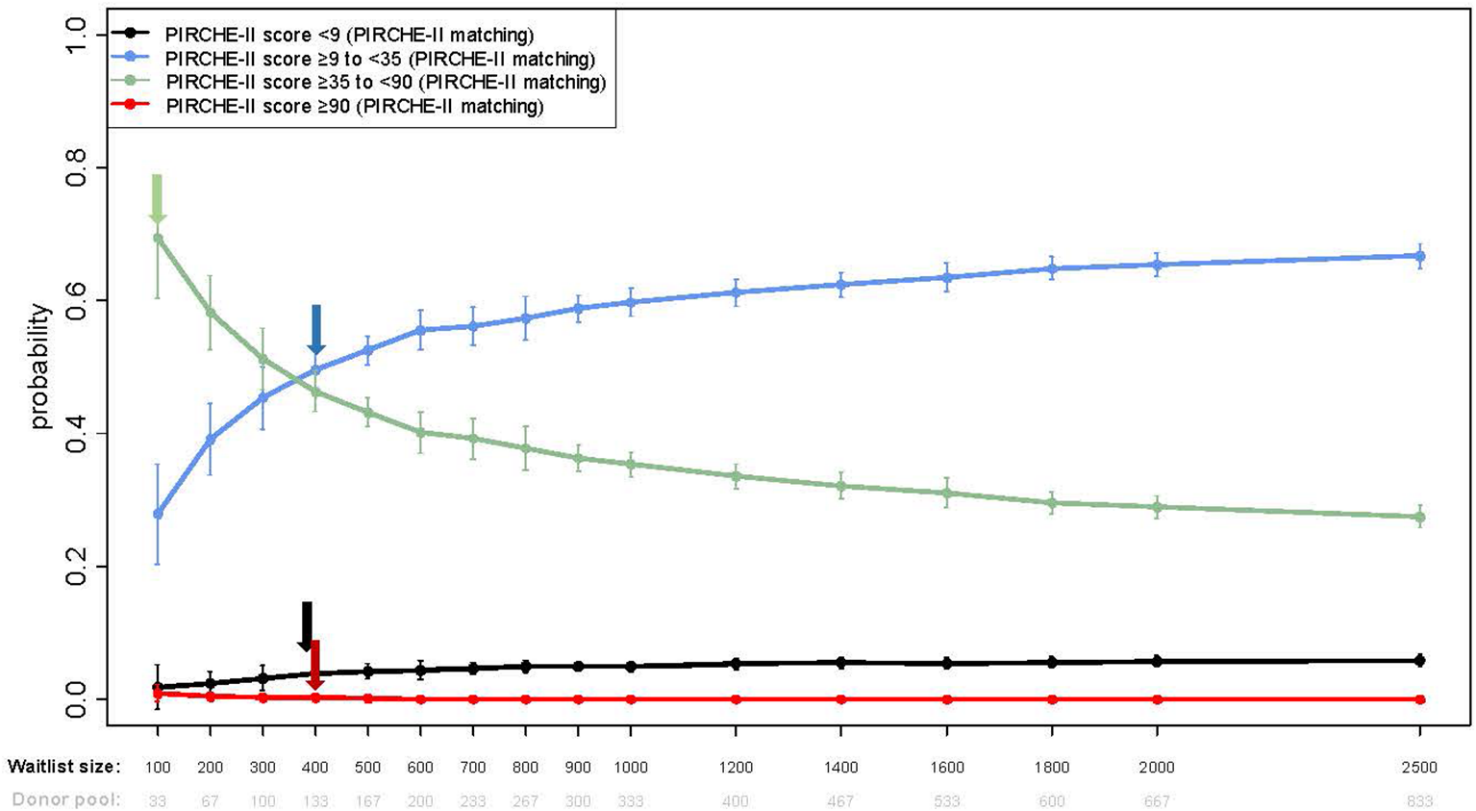
Shown are cumulative probabilities for a set of score thresholds as used in Lachmann et al., 2017 (low (<9), elevated (≥9 to <35), intermediate (≥35 to <90) and high (≥90)) for the simulation results for ABO identical+PIRCHE-matching, a fixed waitlisted candidate-to-donor ratio of 3 and a range of waitlist sizes (from n=100 to 2,500). Error bars are calculated as the standard deviation of 10 repeated simulation runs. Arrow’s indicate the inflection point of the individual cumulative probability curves.

As shown in **Figures 2**, **3**, prospective molecular matching increased the proportion of patients in the lower 2 strata and decreased those in the higher strata, though the allocation ratio was highly dependent on the numbers of patients on the waitlist. The first of these variables is demonstrated in **Figure 3** which shows the cumulative probability of patients dividing into the four PIRCHE mismatch score strata started to saturate at waitlist sizes of n=400 (1.5%) for <9, n=400 (55%) for >9-<35, n=100 (70%) for >35-<90, and n=400 (1%) for >90, respectively (**Figure 3**).

The cumulative probability of achieving a low PIRCHE-II total score of <9 ranged from 1.8% with a waitlist of 100 to 5.8% with a waitlist of 2,500, a small absolute gain. However, the probability of achieving a moderately higher score of ≥9 to <35 more than doubled from 27.9% to 66.7% with corresponding waitlist sizes, a substantial gain. Across this waitlist range, the PIRCHE-II-driven allocation strategy showed a decreasing probability of a score of >35-<90 which declined from 0.694 at n=100 to 0.275 at n=2,500. Very few transplant pairs had a PIRCHE-II score >90 which declined from a probability of 0.009 at n=100 to zero at n=2,500).

**Figure 4:**
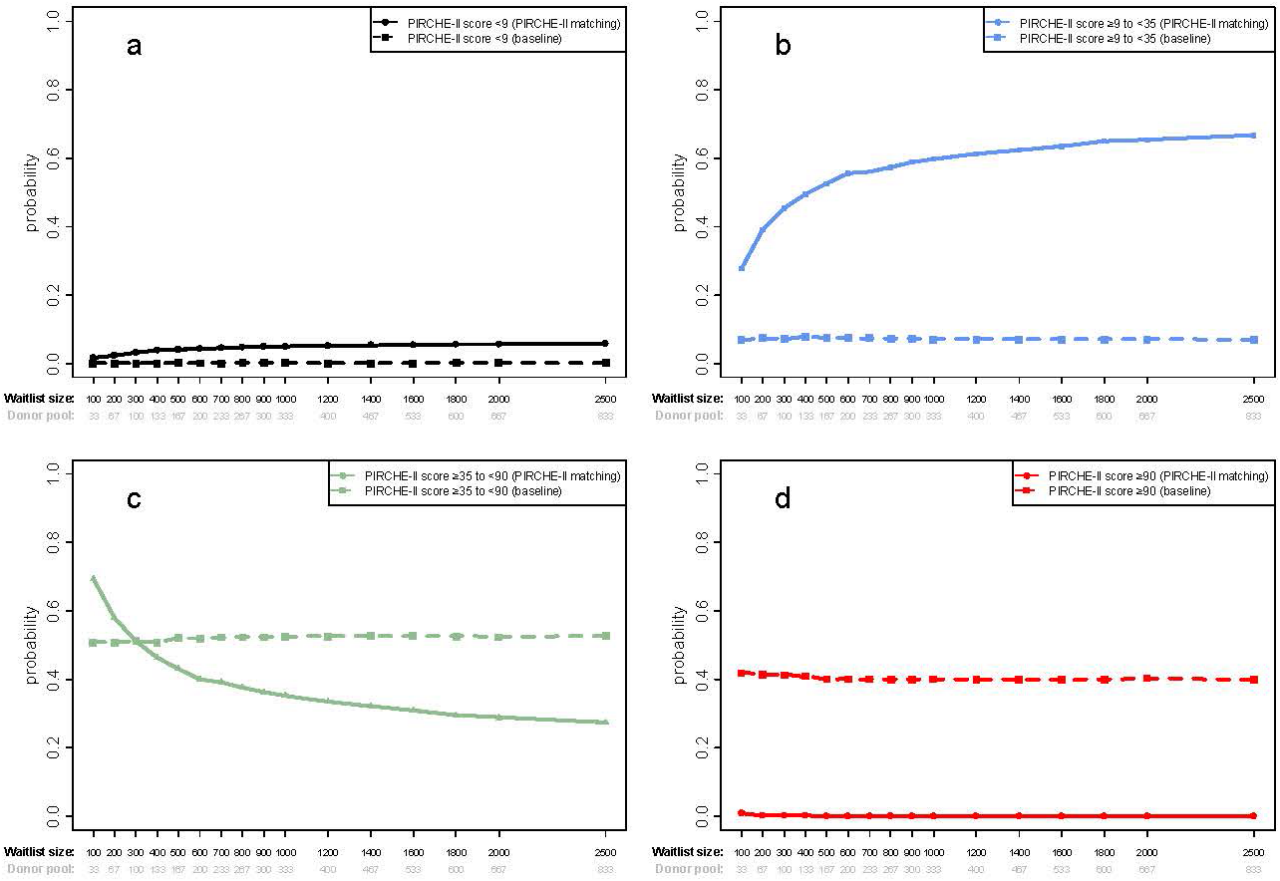
Shown are cumulative probabilities for a set of score thresholds as used in Lachmann et al., 2017 (low (<9) **(a)**, elevated (≥9 to <35) **(b)**, intermediate (≥35 to <90) **(c)** and high (≥90) **(d)**) for the simulation results for ABO identical+PIRCHE-matching (solid line) relative to base-case allocation (dotted line), a fixed waitlisted candidate-to-donor ratio of 3 and a range of waitlist sizes (from n=100 to 2,500). Error bars are calculated as the standard deviation of 10 repeated simulation runs.

Compared to base-case allocations, prospective PIRCHE-II based allocation provides a higher probability of achieving a low PIRCHE match of <9 or a more moderate score of ≥9 to <35 (**Figures 4a, b**), whilst concurrently providing a lower probability of an intermediate (≥35 to <90) or high score (≥90) (**Figures 4c, d**). At a benchmark of average waitlist size (n=290), the fraction of low-risk transplantations increases from 1.4% in base-case allocations to 3.2% when deliberately matched, which does not improve much with growing waitlist size. There is a substantial expansion of the elevated risk category, from 7.7% in base-case allocation to 42.8% of all transplant pairs when striving to minimize the PIRCHE-II score, increasing even further to 65.4% at the national waitlist size (n=2000). While transplantations yielding the intermediate risk group remain similarly frequent - 50.9% in base-case allocation to 53.8% when deliberately matched, the frequency of high-risk transplantations is expected to drop from 41.2% to 0.2% when stiving for better compatibility. At the bench-mark waitlist size (n=290), patients would be equally likely to get an intermediate risk offer (≥35 to <90) with either allocation simulation, which decreases with larger waiting lists using deliberate matching in favor of an increased frequency of the high-risk group (**Figure 3**). Importantly, when using deliberate prospective allocation, nearly no patient would receive a high risk (≥90) donor offer, compared to base-case scenarios, which have a 40% probability of being offered a donor kidney with a high-risk score (**Figure 4d**).

#### Comparison of single gene and multigene decision strategies to optimize PIRCHE-II matching

The preceding data provide evidence to support the effect of prospective matching in improving PIRCHE-II compatibility, but do not provide the granularity required to establish rules for precise selection of target genes and expected matching thresholds. These are more clearly evident in **Figure 2**, which demonstrates the probability of achieving defined PIRCHE-II scores for each individual HLA gene and all 5 genes combined. Base-case frequency distribution curves are shifted to the left by purposeful molecular matching (shown in red). Curves reflecting each of the 5 individual genes reach an asymptote (probability approaching 100% of achieving a specific score) with a PIRCHE-II score of < 10, while this value is substantially higher (>40) when all 5 genes are included in the calculation.

**Figure 5:**
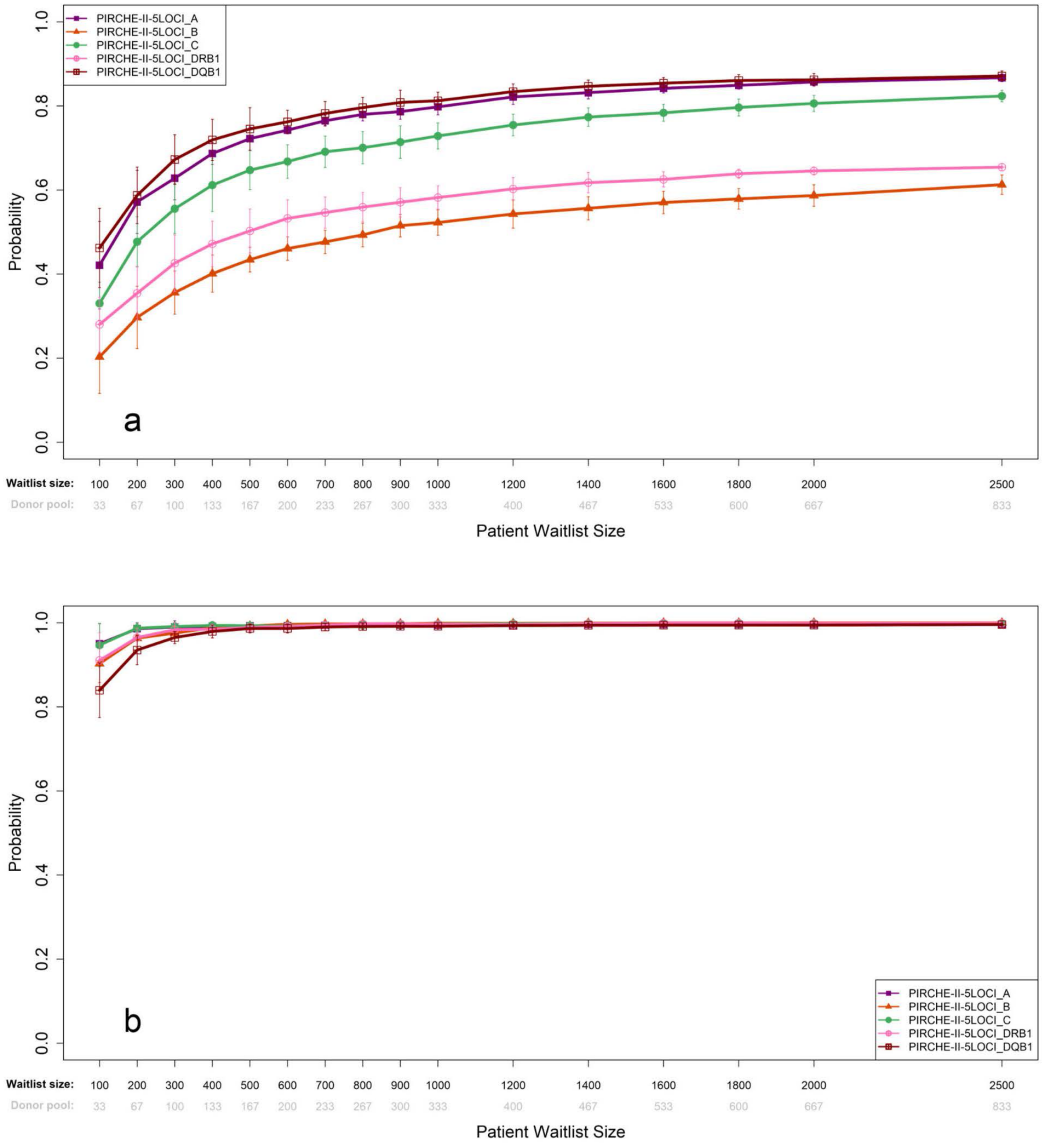
Shown are cumulative probabilities for a score threshold of zero **(a)** and ten **(b)** for the individual genes from the 5LOCI simulations. Results were derived for ABO identical+PIRCHE-matching, a fixed waitlisted canditate-to-donor ratio of 3 and a range of waitlist sizes (from n=100 to 2,500). Error bars are calculated as the standard deviation of 10 repeated simulation runs.

Cumulative probabilities of achieving a zero PIRCHE-II score for each of the five loci are shown in **Figure 5a** and for achieving a score of 10 or less (**Figure 5b**). Waitlist sizes of ~200 would be sufficient to achieve a cumulative probability of matching any of the individual class 1 genes (HLA-A (57%), -B (30%), -C (48%)), while a waitlist size of ~300 would be required to provide 43% and 67% of patients with zero PIRCHE-II donor offers for HLA-DRB1 and -DQB1, respectively.

Smaller waitlist sizes of ~200 would be able to achieve a cumulative probability of between 90% (DQB1)- 100% (HLA-A and -C) of patients receiving a donor offer with a threshold of ≤10 PIRCHE-II score, for any of the individual loci, suggesting that even local waitlist sizes would be able to allocate for optimal PIRCHE-II score (≤10) if only matching for DQB1 (**Figure 5b**).

### Probability of matching varies by patient

#### Flow diagram of patient dynamics

**Figure 6:**
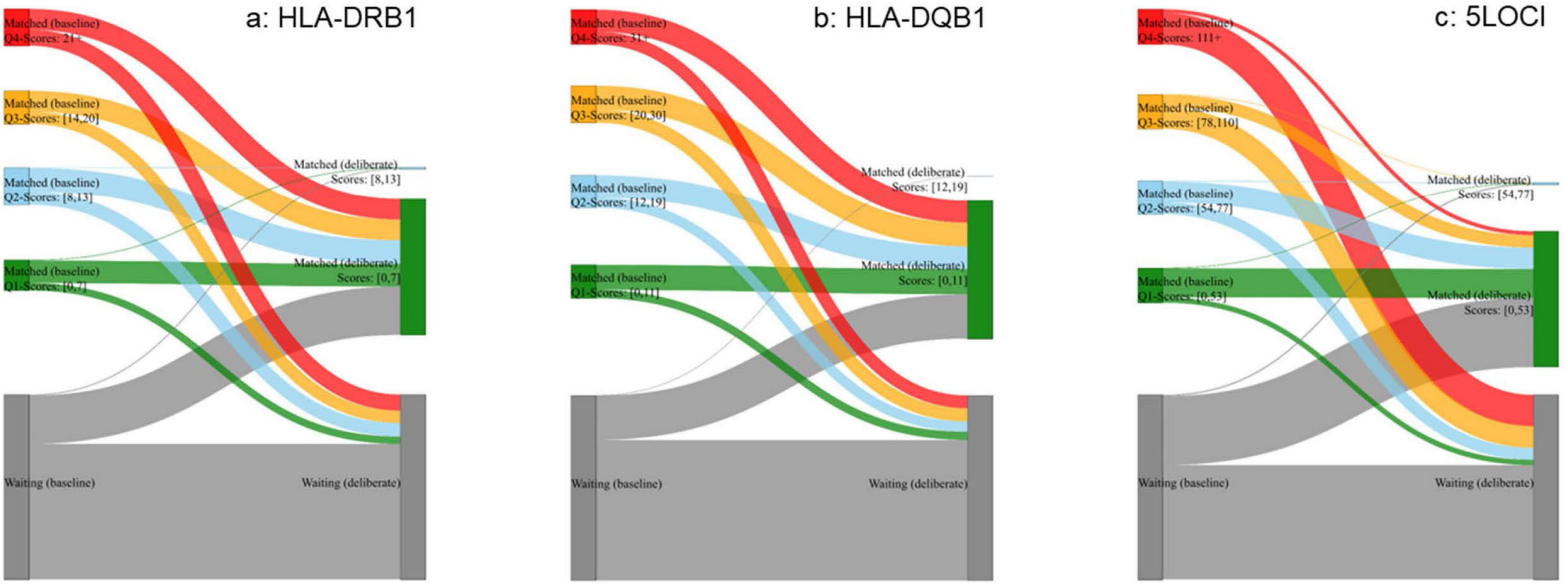
Shown are results for a patient flow analysis for one of the ten repeated simulations for Canada, when matching at individual HLA-DRB1 **(a)**, HLA-DQB1 **(b)** and all 5 HLA genes combined **(c)**. Observed counts in different categories at the end of the simulation are represented as sized, colored boxes for baseline (left) and deliberate PIRCHE-II matching (right). Waitlist size and order of donors and recipients were identical in the two simulations. Patients, and corresponding scores if the patient was matched, were tracked. Flows were calculated and displayed in the corresponding category colour, sized proportionally to absolute count. A ‘Waiting’ category was added to keep track of patients who were still on the waitlist at the end of the simulation. HLA-DRB1 **(a)** corresponding PIRCHE scores for Quartile 1=0-7, Quartile 2=8-13, Quartile 3=14–20 and Quartile 4=>21. 177 of 406 patients in the baseline Q4-score group (left; red box) were not matched in the deliberate matching scenario. The remaining 229 Q4-score patients at baseline all received a Q1-score transplant in deliberate matching. HLA-DQB1 **(b)** corresponding PIRCHE scores for Quartile 1=0-11, Quartile 2=12-19, Quartile 3=20–30 and Quartile 4=>31. 140 of 383 patients in the baseline Q4-score group (left; red box) were not matched in the deliberate matching scenario. The remaining 243 Q4-score patients at baseline all received a Q1-score transplant in deliberate matching. 5LOCI combined **(c)** corresponding PIRCHE scores for Quartile 1=0-53, Quartile 2=54-77, Quartile 3=78–110 and Quartile 4=>111. 346 of 389 patients in the baseline Q4-score group (left; red box) were not matched in the deliberate matching scenario. The remaining 43 Q4-score patients at baseline all received a Q1-score transplant in deliberate matching.

Additionally, we investigated individual patient dynamics within the simulated scenarios, to see if the allocation models benefited or disadvantaged particular subsets of patients. A patient- flow analysis (**Figure 6**) compared scores achieved for baseline and deliberate matching scenarios. Patients and donors entered the simulation in exactly the same order. 1,542 organs from 762 donors were matched, but not necessarily the same patients in baseline and deliberate simulations. Patients not transplanted at the end of the simulation (grey category) remained on the waitlist. Baseline match-scores were used to define quartile score categories and the score-ranges were used to define categories for the deliberate simulation match scores. Patients in the two simulations could be traced from baseline to deliberate as shown in **Figures 6a–c** for DRB1, DQB1 and 5LOCI scores. For DRB1 and DQB1, the majority of patients with baseline scores in the Q1, Q2, Q3 and Q4 categories, as well as a proportion of waitlist patients, were allocated a lower PIRCHE score (green bar on the right), while for 5LOCI, the majority of patients with baseline scores in the Q3 and Q4 categories were not transplanted in the deliberate matching scenario.

#### Simulation shows that probability of matching varies by patient

**Figure 7:**
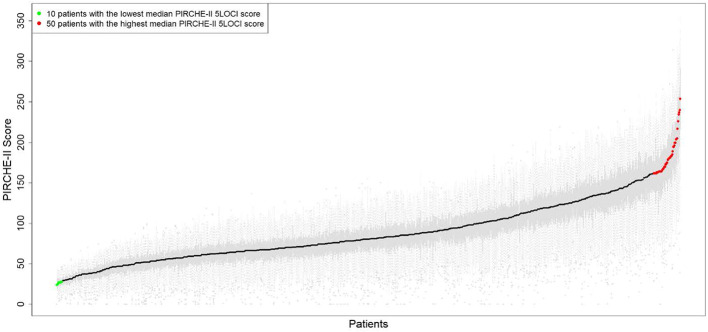
The static PIRCHE-II score (y-axis) distribution of all 1,150 patients (x-axis) against ABO-matched donors shows distributions as grey boxplots with black median scores overlaid. Whiskers extend to the most extreme data point which is no more than 1.5 times the interquartile range from the box. The median scores range from 24 (leftmost patient) to 254 (right-most patient). Individual scores over all identical ABO-matched pairs ranges from 0 to 357. Results are for the PIRCHE-II 5LOCI scores. A demonstrable increasing slope towards the right of the curve highlights a small cluster of patients (indicated in red, n=36, 3.13%) who had a smoothened PIRCHE−II score difference (k=±5 window) >0.508 (0.2% of maximum median score of 254).

The static PIRCHE-II score distribution of all 1,150 patients (x-axis) against ABO-matched donors shows distributions as grey boxplots with black median scores overlaid (**Figure 7**). Recipients are ordered by median score demonstrating that variation around the median increases as median scores increase. A demonstrable increasing slope towards the right of the curve highlights a small cluster of patients (indicated in red, n=36, 3.13%) who had high median PIRCHE−II score, for all matches, suggesting this subset of patients would be less likely to obtain a low-risk match.

## Discussion

Advances in gene sequencing now allow clinical HLA laboratories to define HLA genes at high resolution in just a few hours, achieving single-base resolution genotypes within the limited time frame of donor offers (~6hrs) (31). And HLA structural biology has provided further insight into the mechanism of immune recognition of donor graft antigens, highlighting the fact that reducing HLA disparity plays an important role in patient outcomes (7–9, 32). This suggests that precise HLA compatibility is an attainable goal for organ allocation. However, although retrospective studies have demonstrated an association between high molecular compatibility and improved outcome (22, 25, 33, 34), the question remains unresolved until the feasibility of prospectively allocating organs based on HLA-molecular matching is established.

To provide insight into this, we present simulations using a highly-defined provincial population to model feasibility of prospectively matching for optimal PIRCHE-II score, albeit with a minimal, base-case allocation match rule. This is a necessary initial preliminary exploration of transition from a queue-based (or waiting-time-based) allocation to a utility-based allocation, and provides valuable data needed to inform strategic decisions for incorporating PIRCHE-II scores into allocation policy and clinical practice. We have previously modelled allocation feasibility incorporating HLAMatchmaker B-cell eplets (29), and in this study we focus on T-cell epitopes, as predicted by the PIRCHE-II algorithm (19) which employs computationally predicted theoretical peptides derived from mismatched donor HLA molecules presented by recipient DRB1 molecules as part of indirect presentation to T-cell receptors leading to activation required for alloreactive responses (*reviewed in* (35).

The current study suggests that optimizing organ allocation to achieve lower PIRCHE-II scores is dependent on the number of genes included, and is further constrained by the numbers of both patients and donors where success is higher with larger waitlists. In our cohort, whilst achieving low 5LOCI PIRCHE-II scores (i.e. <35) is challenging, high compatibility is achievable on single loci scores (for example, for DQB1 or DRB1). Conversely, high-risk 5LOCI PIRCHE-II scores (≥35) are rare, and almost nonexistent when a locus-specific approach is employed. Our data suggest that although full 5LOCI PIRCHE-II matching may not be possible for most patients, prioritizing class 2 loci (DRB and DQB, loci most commonly correlated with post-transplant anti-HLA antibody formation) in allocation strategies can minimize high-risk pairings, even in smaller renal transplant programs. Extending these models to other organs suggests that regional or national sharing may be necessary to optimize PIRCHE-II score for heart, lung and liver transplants whose national waitlists (n=178, 303 and 497) and donor totals (n=141, 306 and 430) are smaller than those of kidney waitlists (30).

Whilst simulated allocation models to include PIRCHE-II score have previously been published (27), the EuroTransplant study sampled from virtual populations to model recipients and donors. Our study uses real-world high-resolution HLA genotypes of donors and candidates from BC with simulations illustrating implications of various waitlist sizes in Canadian populations. It provides one possible approach of including PIRCHE-II metrics into a basic version of local kidney allocation policies. The simulations demonstrate upper boundaries of histocompatibility to be expected in a real world-sized waitlist and provide data suggesting that molecular matching, using limited gene matching, (specifically DRB and DQB may be feasible to realize the advantages of improved compatibility for the majority of patients. It should also be noted that PIRCHE-II scores only become incrementally useful in programs that have an HLA typing resolution appropriate for PIRCHE-II score determination. Low resolution typings (i.e. serology, as in (33)) necessitate imputation of 2-field typings, which, while large accurate (37, 39), become less reliable in smaller and more diverse populations with limited haplotype frequency data (38). Advances in high-resolution HLA genotyping, now more accessible and cost-effective, should be considered for any program integrating PIRCHE-II scores into allocation algorithms (31, 40–42). Our study builds on previous simulated allocation models (27), using real-world high-resolution HLA genotypes from BC to explore the impact of various waitlist sizes within Canadian populations. The simulations define upper boundaries of histocompatibility within a real-world waitlist and provide evidence that molecular matching, specifically prioritizing DRB and DQB, may be a viable strategy to enhance compatibility for the majority of patients.

Our study has limitations that we are actively addressing. Conducted in a nested group within a single provincial program, representativeness may be a concern, though BC has one of the most ethnically diverse populations in Canada. We are engaged with further studies to validate these assumptions, by expanding to larger validation cohorts within national datasets. Our allocation models are also intentionally limited to ABO-identical matching (to mimic current Canadian rules) and optimal PIRCHE-II scoring. Our simplified allocation strategy may also underrepresent challenging-to-match patients, due to the short, one-year time horizon used in these simulations. While retrospective studies suggest benefits, integrating molecular-compatibility metrics into existing allocations systems is challenging due to the need to maintain balance in factors such as wait time, medical urgency and utility. We are conscious that critical ethical and equity concerns have not been tackled in this current study. The GCTC team have previously reported strong public support for molecular matching and proposed countermeasures to facilitate implementation (36). In reality, countermeasures likely need to be incorporated, e.g. a match-probability score (as employed by ET or suggested by the ETKASPIR sim study) for those patients we know a-priori will be challenging to optimize for excellent histocompatibility. Additionally, not all patients benefit from improved histocompatibility by identical pathways. Existing allocation models, such as Eurotransplant’s Senior Program and the UK’s age-weighted histocompatibility scoring (55, 56), reflect this nuance. However, even in older patients, molecular matching may allow for reduced immunosuppression, offering potential advantages of lower toxicity.

Our understanding of immunogenicity of different epitopes remains limited (26, 43–49). Molecular matching is not in its final form, some improvements were already incorporated (e.g. binding promiscuity correction/ranking for PIRCHE), some are planned (e.g. immunogenicity studies) and there are various methods of defining the molecular entity to match on e.g. HLA antigen, PIRCHE, HLA Matchmaker (Eplets), HLA-EMMA (Epitope MisMatch Algorithm), Snow, electrostatic mismatch (EMS), hydrophobicity mismatch (HMS), amino-acid mismatch (AAMM). The approach of how to perform molecular matching is still under debate (45, 50–52), but it is generally accepted that better matching – independent of allorecognition pathway – does associate with improved outcome (50, 51). A minority of patients are unable to obtain a high degree of molecular compatibility based on e.g. rare haplotypes, admixed populations or infrequent ancestry-representation in the donor pool. These patients will need careful consideration to account for equity issues. Studies are currently underway to review this and evaluate the use of a PIRCHE-II risk profile score which reflects that it may not be feasible to find low PIRCHE-II score donors for every patient (30).

This study does not propose the presented simplified method as a recommended allocation policy but rather explores the underlying concept. The model does not account for key limitations or current Canadian allocation priorities, such as pediatric recipients and sensitized patients with pre-formed antibodies, both of which would further restrict the available donor pool and reduce matching opportunities. The primary objective was to assess whether this approach warrants further investigation, recognizing that significant knowledge gaps remain, and that substantial work is needed before any consideration of implementation. National organ sharing programs must also consider key operational constraints, such as cold ischemia time (especially for thoracic organs), geographical constraints and shipping logistics, which are not accounted for in this analysis. These gaps are being addressed as part of a larger Genome Canada research program (53), including addressing key allocation determinants to fully model true feasibility. Stringent avoidance of recipient pre-sensitization has radically reduced the incidence of AMR within this program, and improved molecular compatibility between donors and recipients offers the potential to further reduce rejection and improve long-term survival. These data will now be considered within the next phase of this program which will implement a national prospective molecular compatibility strategy to achieve these goals.

## Data Availability

The datasets presented in this article are not readily available because of clinical confidentiality, the dataset employed in this article comprising of patient data collected and generated for this study. Data may be requested through the corresponding author under a formal data sharing agreement. Requests to access the datasets should be directed to Paul.Keown@ubc.ca.

## Supplementary material

Supplementary Table 1 HLA alleles and genotypes of donor and patient baseline cohorts (identical genotypes are only counted once).

Supplementary Table 2 Demographics of donor and patient baseline cohorts.
