# Supplementary material for: Simulation of prospective PIRCHE-II molecular matching in Canada: a feasibility study
Source: Front Immunol. 2026 Feb 10;17:1703762. doi: 10.3389/fimmu.2026.1703762 (PMC12929530; doi:10.3389/fimmu.2026.1703762)
Supplement: Supplementary Table 1 — HLA alleles and genotypes of donor and patient baseline cohorts (identical genotypes are only counted once). [file Table1.pdf]

Supplemental Table 1. HLA alleles and genotypes of donor and patient baseline cohorts (identical genotypes are only counted once).

Genes	Alleles	Genotypes
All 11 Genes	398	1404
Class I	223	1270
Class II	175	1265
A	64	252
B	107	570
C	52	251
DQA1	22	119
DQB1	19	116
DPA1	7	15
DPB1	47	187
DRB1	62	379
DRB3	8	15
DRB4	5	10
DRB5	7	14
DRB1/3/4/5	80	517
DRB1/3/4/5 + DQB1	99	651
