# Supplementary material for: Simulation of prospective PIRCHE-II molecular matching in Canada: a feasibility study
Source: Front Immunol. 2026 Feb 10;17:1703762. doi: 10.3389/fimmu.2026.1703762 (PMC12929530; doi:10.3389/fimmu.2026.1703762)
Supplement: Supplementary Table 2 — Demographics of donor and patient baseline cohorts. [file Table2.pdf]

Supplemental Table 2. Demographics of donor and patient baseline cohorts.

	**DD**<br>N = 261	**Patients**<br>N = 1,150
**Blood Group, n (%)**		
A	94 (36%)	406 (35%)
AB	8 (3.1%)	45 (4%)
B	32 (12%)	192 (17%)
O	127 (49%)	507 (44%)
**Sex, n (%)**		
F	54 (21%)	442 (38%)
M	80 (31%)	707 (38%)
Unknown	127 (49%)	1 (0.1%)
**Age, Mean (SD), [Range]**	42 (15), [7 - 73]	54 (16), [1 - 83]
Unknown	37	0
